# A 17 year old male with a testicular fibrothecoma: a case report

**DOI:** 10.1186/1746-1596-8-152

**Published:** 2013-09-17

**Authors:** Michael-Wadih Sourial, Robert Sabbagh, Alexandre Doueik, Yves Ponsot

**Affiliations:** 1 Surgery Department, Urology Division, Faculté de Médecine et des Sciences de la Santé de l’Université de Sherbrooke, Centre Hospitalier Universitaire de Sherbrooke, Sherbrooke, Québec; 2 Pathology Department, Faculté de Médecine et des Sciences de la Santé de l’Université de Sherbrooke, Centre Hospitalier Universitaire de Sherbrooke, Sherbrooke, Québec

**Keywords:** Fibrothecoma, Sex cord stromal tumors

## Abstract

We herein report the case of a right-sided testicular fibrothecoma in a 17 year old male and review the pertinent literature relatable to this rare, benign lesion.

**Virtual slides:**

The virtual slide(s) for this article can be found here: http://www.diagnosticpathology.diagnomx.eu/vs/7738283021019280.

## Background

Sex cord/stromal tumors (SCSTs) are rare, comprising approximately 4–5% of all gonadal neoplasms that originate from the gonadal matrix. The term refers to neoplasms containing Leydig cells, Sertoli cells, granulosa cells, or theca cells [1]. In females, thecomas are tumors that arise from the hormone-secreting theca cells that surround the ovarian follicles. They are relatively common SCSTs, typically presenting in post-menopausal women in their mid-60s. They are some of the most hormonally active tumors of all SCSTs and usually present with signs or symptoms of excess estrogen such as abnormal vaginal bleeding, endometrial hyperplasia or adenocarcinoma [2]. Bilateral or extragonadal disease is extremely rare. These tumors are clinically benign and their surgical resection is curative. In males however, only a few case reports of theca cell tumors have been reported, and only one case report of a pure fibrothecoma has been reported. We report what to our knowledge is the second case of pure fibrothecoma.

## Case presentation

**Figure 1:**
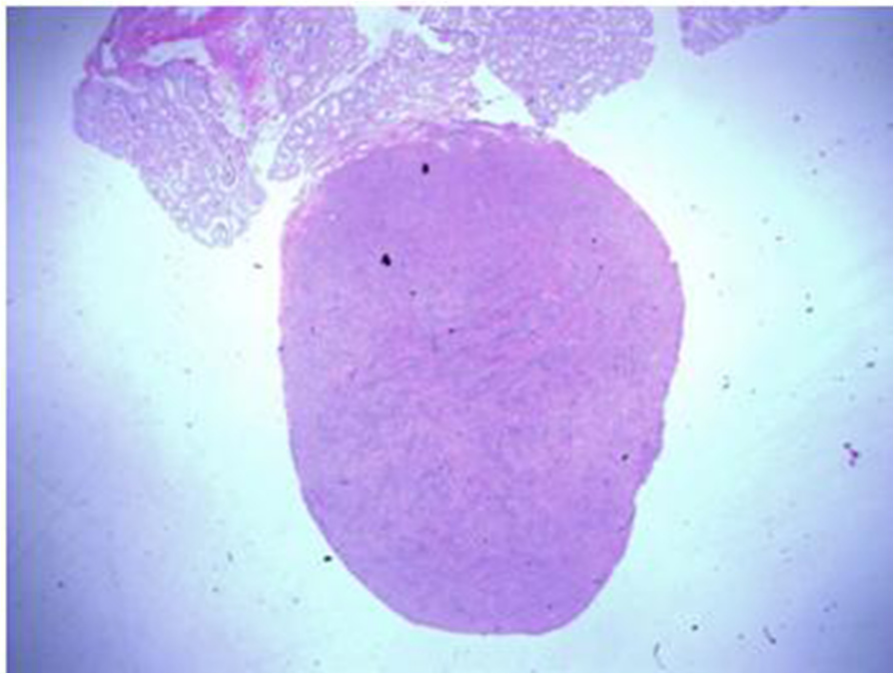
Fibrothecoma, note the well-circumscribed lesion, non-adherent to adjacent testicular parenchyma.

**Figure 2:**
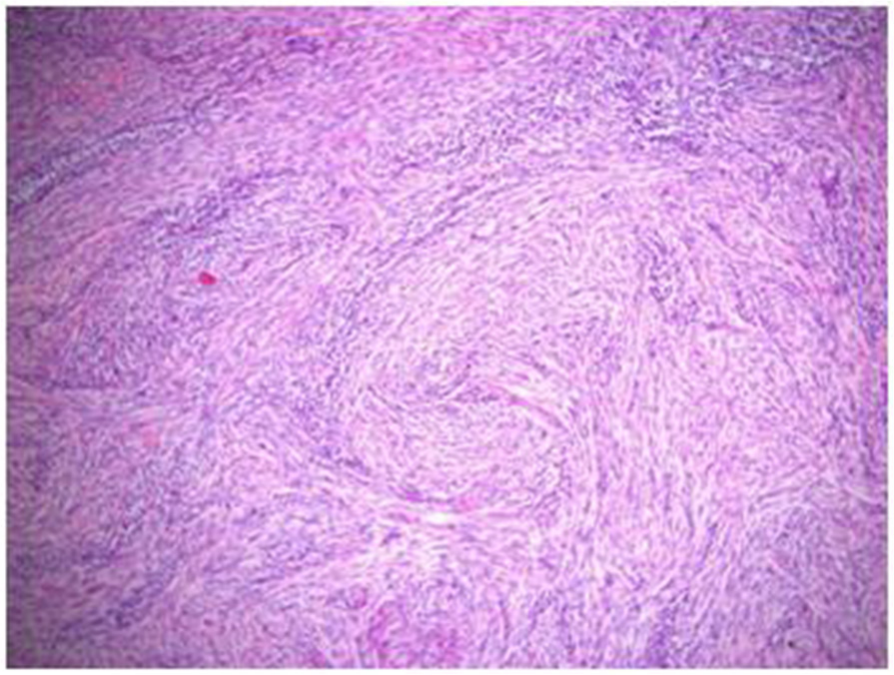
Fibrothecoma, H&E stain.

A seventeen year old otherwise healthy male was referred to us for a palpable, painless right-sided testicular mass. He had a normal physical appearance with no apparent dysmorphisms or signs of feminization, and was evaluated a Tanner stage V. Testicular ultrasound showed a hypoechogenic solitary 8 mm solid right intratesticular mass of undetermined etiology with no associated hydrocele. The left testicle was normal. Pre-operative testicular markers were negative as follows: ß-HCG < 1 (normal 0–5 mIU/mL), AFP 1.2 (normal < 7 ng/mL), and LDH 179 (normal 120–250 IU/L). Sex hormones were not obtained. We carried out a right radical inguinal orchiectomy on the suspicion of a malignant tumor and the patient was discharged the same day with no early or late complications. Post-operative computed tomography (CT) scans of the thorax, abdomen and pelvis showed absence of retroperitoneal adenopathies or any suspicious metastatic lesions. Final pathology report described a 9 × 9 × 9 mm whitish-beige mass that was well-circumscribed and non-adherent to the adjacent testicular parenchyma (Figure 1). There was no lympho-vascular invasion, invasion of the tunica albuginae, invasion of the rete testis or epididymis, or any other testicular lesions. The morphologic appearance and immunohistochemical profile suggested a testicular fibrothecoma (Figure 2). Margins were negative.

## Discussion

Sex cord/stromal tumors are rare, comprising only 4–5% of all gonadal neoplasms. By definition, the term SCSTs refers to neoplasms containing Leydig cells, Sertoli cells, granulosa cells, or theca cells. The scarcity of these tumors limits our understanding of their natural history, treatment, and prognosis. The clinical and scientific significance of this case presentation is to increase awareness of the occurrence of this rare entity, especially to practicing urologists and pathologists, and also to review its clinical and pathologic presentation, as part of a differential diagnosis for a testicular tumor.

In females, fibrothecomas typically present in post-menopausal women in their mid-60s, and very rarely present in women less than 30 years of age [3]. In males, only four case reports of testicular fibrothecoma have been reported. Van der Horst et al. [4] reported the first and only case of a pure fibrothecoma in a 54 year old male presenting with intermittent pain in his left testis.

Clinical presentation of this rare entity will depend on the age and sex of the patient. Theca cells are stimulated by luteinizing hormone (LH) and secrete androstenedione, which is uptaken by the adjacent granulosa cells and converted into estradiol. In females, excess estrogen may lead to abnormal vaginal bleeding, endometrial hyperplasia or endometrial adenocarcinoma [2]. Occasionally the thecomas are luteinized and may secrete androgens and even potentially induce masculinization. In children, they can present as precocious sexual puberty evidenced by prominent external genitalia, pubic hair growth or masculine voice. Males with these tumors may present with signs and symptoms of estrogen or androgen excess, including gynecomastia, impotence or decreased libido. The most common presentation in males remains a painless testicular mass.

**Figure 3:**
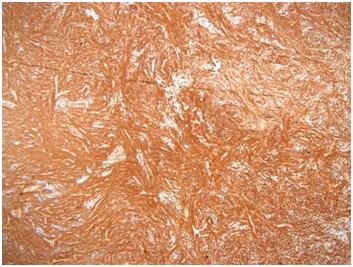
Fibrothecoma, smooth muscle actin stain.

**Figure 4:**
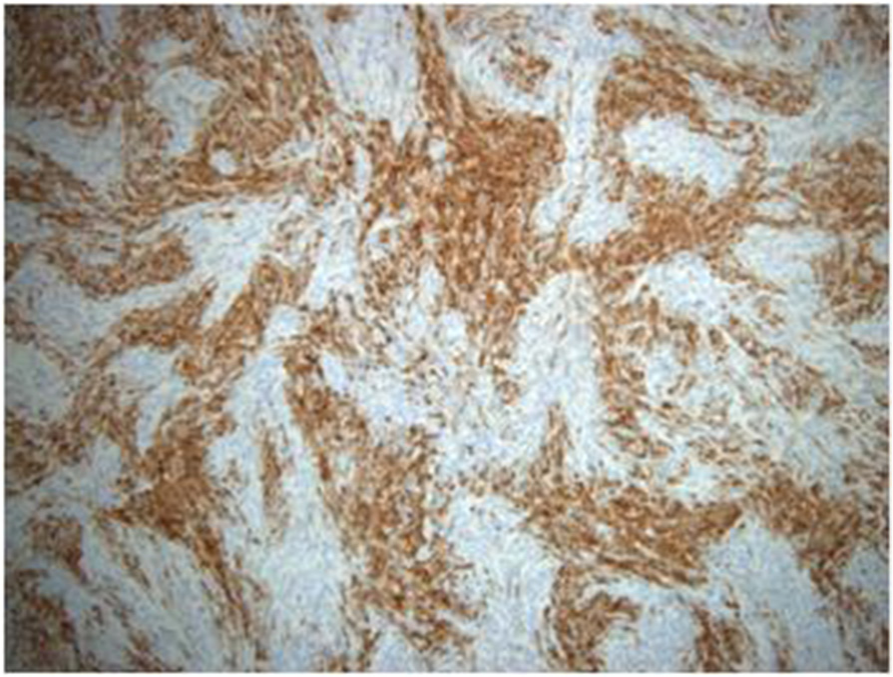
Fibrothecoma, inhibin stain.

Typical cell type findings include spindle-shaped cells without any formally identifiable Sertoli, Leydig, or granulosa cell component. Fibrothecoma tumors are usually well delineated, highly cellular, monotonous, and without any cytological atypia. Sometimes these tumors contain acellular fibrohyalinized zones. Mitotic activity is typically reduced. Immunohistochemistry analysis will show that the spindle-shaped cells express positivity for smooth muscle cells marker, smooth muscle actin (Figure 3), CD99, PS100, inhibin (Figure 4), and keratins.

It has been postulated that ovarian fibrothecomas are etiologically associated with a tumorigenic syndrome called *nevoid basal-cell carcinoma* syndrome, also known as Gorlin syndrome, or abnormalities in the candidate gene, *PTCH*. Gorlin syndrome is an autosomal dominant syndrome that presents with neurological and musculo-skeletal anomalies, unusual facial appearance, and a higher risk of basal cell carcinoma at or around puberty. Ueda et al. [5] have reported the first case of a testicular thecoma in an 11 year old boy with Gorlin syndrome.

Schenkman et al. [6] reported a case of synchronous bilateral testicular tumors of different histological types, with simultaneous appearance of a mixed non-seminomatous germ cell tumor of the left testis and theca cell tumor of the right testis in a 34 year old male. Eble et al. [7] reported the only known case to date of a metastatic SCST of the testis. The primary lesion was mixed and consisted of granulosa cells, theca cells, Sertoli cells and undifferentiated gonadal stroma. Metastasis occurred in the retroperitoneal lymph nodes and was confirmed by biopsy which showed a Sertoli cell predominance. The patient died 13 months after orchiectomy.

Diagnostic evaluation includes laboratory testing and imaging. Elevated levels of testosterone or estrogen may be seen in secreting tumors, but these are typically not obtained pre-operatively because the diagnosis of SCST is not suspected [3]. The common testicular tumor markers LDH, AFP and hCG are typically normal in SCSTs. Imaging in any male presenting with a testicular mass, or unexplained testicular signs or symptoms should always include testicular ultrasonography.

In the majority of cases, surgical resection of SCSTs is the primary treatment and is most often curative. Fibrothecomas are clinically benign and are usually confined to the gonads at the time of clinical presentation. With early-stage detection and complete excision of the lesion, the overall prognosis is excellent.

## Conclusion

We present what to our knowledge is the second case of a pure fibrothecoma in a male. Fibrothecomas are a distinct clinical and pathologic entity among sex cord/stromal tumors, and awareness of this rare entity is especially important for practicing pathologists and urologists.

## Consent

Written informed consent was obtained from the patient as well as the patient’s parents for the publication of this report and any accompanying images.

## Competing interests

The authors declare that they have no competing interests.

## Authors’ contributions

MS, RS, and YP were all involved in writing and/or revision of the manuscript. YP was the surgeon who proceeded with the orchiectomy. AD was involved in the pathological analysis and contributed to the pathological description of fibrothecomas written in the manuscript. All authors read and approved the final manuscript.
